# Anthropomorphism-based causal and responsibility attributions to robots

**DOI:** 10.1038/s41598-023-39435-5

**Published:** 2023-07-28

**Authors:** Yuji Kawai, Tomohito Miyake, Jihoon Park, Jiro Shimaya, Hideyuki Takahashi, Minoru Asada

**Affiliations:** 1 Symbiotic Intelligent Systems Research Center, Institute for Open and Transdisciplinary Research Initiatives, Osaka University, Suita, Osaka 565–0871 Japan; 2 Department of Adaptive Machine Systems, Graduate School of Engineering, Osaka University, Suita, Osaka 565–0871 Japan; 3 Center for Information and Neural Networks, National Institute of Information and Communications Technology, Suita, Osaka 565–0871 Japan; 4 Department of Systems Innovation, Graduate School of Engineering Science, Osaka University, Toyonaka, Osaka 560–0043 Japan; 5 International Professional University of Technology in Osaka, Kita-ku, Osaka 530–0001 Japan; 6 Chubu University Academy of Emerging Sciences, Kasugai, Aichi 487–8501 Japan

**Keywords:** Human behaviour, Information technology

## Abstract

People tend to expect mental capabilities in a robot based on anthropomorphism and often attribute the cause and responsibility for a failure in human-robot interactions to the robot. This study investigated the relationship between mind perception, a psychological scale of anthropomorphism, and attribution of the cause and responsibility in human-robot interactions. Participants played a repeated noncooperative game with a human, robot, or computer agent, where their monetary rewards depended on the outcome. They completed questionnaires on mind perception regarding the agent and whether the participant’s own or the agent’s decisions resulted in the unexpectedly small reward. We extracted two factors of Experience (capacity to sense and feel) and Agency (capacity to plan and act) from the mind perception scores. Then, correlation and structural equation modeling (SEM) approaches were used to analyze the data. The findings showed that mind perception influenced attribution processes differently for each agent type. In the human condition, decreased Agency score during the game led to greater causal attribution to the human agent, consequently also increasing the degree of responsibility attribution to the human agent. In the robot condition, the post-game Agency score decreased the degree of causal attribution to the robot, and the post-game Experience score increased the degree of responsibility to the robot. These relationships were not observed in the computer condition. The study highlights the importance of considering mind perception in designing appropriate causal and responsibility attribution in human-robot interactions and developing socially acceptable robots.

## Introduction

Social robots who work with humans are gradually becoming common in our daily lives, where, for example, robots interact with humans as sales recommenders in shops^1^, or assemble and carry products as collaborators in factories^2^. When such human-robot interactions result in an undesirable outcome, how do people subjectively attribute responsibility for it? It is not always clear whether a human or robot was the cause of a failure in interactive situations. Nevertheless, a person will sometimes infer a cause and attribute responsibility to somebody or something for the failure, as is the case in the human-human situations^3^. To develop a socially acceptable robot, it is important to clarify the psychological processes of causal and responsibility attributions in human-robot interactions.

Attribution theory in interpersonal relationships is well-established in social psychology^4,5^. Humans estimate the cause of an event or action, and then attribute responsibility (e.g. blame or credit) for an outcome of the event based on the estimated cause. Many studies have shown that such an interpersonal attribution process can be applied to attribution to machines (e.g.^6–8^). Specifically, it has been reported that a human’s self-serving bias that leads one to view the cause and responsibility for a negative outcome as not attribute to one’s self^9^ can be observed even in human-machine interactions^6,7,10–13^.

Behind the similarities in attributions to humans and machines, there seems to be a process by which machines are anthropomorphized^14^. The “mind perception” scale was proposed by Gray et al.^15^ to evaluate anthropomorphized mental capabilities for several agents including humans, robots, and computers. Dimensionality reduction revealed two orthogonal dimensions of mind perception: the “Experience” dimension, representing the abilities to sense and feel emotions, and the “Agency” dimension, representing the abilities to plan and execute intentional actions. Takahashi et al.^16^ showed that the Experience and Agency dimensions for non-living agents correspond to “emotion” and “intelligence,” respectively.

We aimed to clarify the process from mind perception to causal and responsibility attributions when it is unclear whether the cause of a failure is a human or agent. In our experiment, a human participant and a partner agent conducted a non-cooperative repetitive game where they received a monetary reward based on both of their decisions. We designed the agents’ behavior so that the participants received an unexpectedly small sum of money, thus failing at the game. The participants then answered questions about causal and responsibility attributions for the failure and their mind perception about the agent. To clarify the different attribution processes among the agents, we set three agent conditions that have different levels of anthropomorphism: human, robot, and computer. We extracted the Experience and Agency dimensions and analyzed correlations between these scores and the degrees of causal and responsibility attributions for each agent condition. Further, using SEM analysis, we constructed the best fit models from mind perception to causal and responsibility attributions for each agent type. We verified the hypotheses that are introduced in the next section and examined different attribution processes among the agents by combining the correlation and SEM analysis results.

### Related studies

A survey reported that most respondents perceived agency (i.e. mental capabilities including thought and decision-making) in computers, and some respondents presumed a computer to be responsible for errors in practical scenarios^17^. Further, some studies on human-robot interactions showed a robot’s appearance and behavior can bias the attribution process. Hinds et al.^7^ found that a human-like robot was blamed for failure in a task more than a machine-like robot. Kim and Hinds^18^ further demonstrated that more blame or credit for a task outcome was attributed to an autonomously behaving robot than a non-autonomous robot, even if the autonomous behavior did not directly contribute to the task. These studies suggest that humans may attribute responsibility to a computer or robot based on the machines' anthropomorphized mental capabilities (e.g. perceived agency and autonomy). However, these existing studies did not quantify the types and degrees of the anthropomorphism to investigate a direct relation between mental attribution and responsibility attribution. There are various types of minds that are anthropomorphically attributed to nonhumans, including emotional and intellectual capabilities. Therefore, in the current study, we quantify anthropomorphism using the mind perception scale to investigate the relationship between Experience and Agency scores and causal and responsibility attributions.

Gray et al.^15^ proposed the mind perception scale and demonstrated that Agency scores strongly correlate with the degree of responsibility attribution. They showed participants pictures of two agents and asked them “if both characters had caused a person’s death, which one do you think would be more deserving of punishment?” As the result, people attributed more responsibility to agents with higher Agency. van der Woerdt and Haselager^8^ also showed that agency perception regarding a robot would relate to increased blame for that robot’s failure. These studies suggest that people hold a belief that agents with agency capabilities should be responsible (i.e. blamed or punished) for their actions. However, if the cause of the failure is vague, and a human evaluator must infer which agent (e.g. the evaluator’s self or the robot) was the cause, he or she might find it hard to believe that an intelligent robot caused the failure. Therefore, agency perception regarding the robot might decrease the degree of causal attribution to it.

Our previous study investigated the relationship between mind perception and causal attribution of game failures, in which the cause was vague^19^. Experiments were conducted with three partner agents: a human, robot, and computer. We found that regardless of the type of agent, Agency scores had a negative correlation with the degree of causal attribution. This result implies that more intelligent agents are less likely to be attributed causes. However, the study did not consider responsibility attribution which is a distinct concept from causal attribution. While causal and responsibility attributions are closely related, i.e. those who caused a failure should be responsible for it, various factors including intentionality and emotional biases affect responsibility attribution^4,5,20^. Considering the emotional biases, Experience may be also important in responsibility attribution. In the current study, we use the same game design as the previous study^19^ and add a question about responsibility attribution to investigate the relationships between causal and responsibility attributions and mind perception. Further, SEM analysis is performed to develop models that simply explain the relationships among many of these variables.

## Hypotheses

Existing studies^8,15^ have proposed that a robot’s agency as perceived by a human evaluator leads to more responsibility attribution to the robot when the robot evidently causes an undesirable outcome. However, if it is unclear whether the evaluator’s self or the robot is the cause of the outcome, does the robot’s perceived agency increase the degree of causal attribution made to the robot? Instead, the evaluator might attribute the cause to him or herself rather than to the intelligent robot with a high level of agency. To clarify these processes, we hypothesized the following:

### Hypothesis 1

Agency decreases the degree of causal attribution to an agent.

Responsibility is attributed to an actor if an evaluator thinks the actor could have foreseen and intentionally caused an action’s outcome^4,5^. Therefore, a key factor in the responsibility attribution process is the inference of an actor’s intentions and motivations. Additionally, this process is biased by the evaluator’s emotional response to the actor; for example, an unfavorable impression may lead to more responsibility attribution to an actor^20^. This suggests that an agent’s Experience in mind perception, which indicates emotional capabilities, might give an evaluator the unfavorable impression that the inappropriate action was intentional and motivated by the agent’s emotions. This impression might result in more responsibility attribution, which leads to the following hypothesis:

### Hypothesis 2

Experience increases the degree of responsibility attribution to an agent.

Existing studies on attribution to machines did not consider changes in impressions during a task. However, the adaptation gap hypothesis states that the difference between a user’s expectation and the actual performance of an agent strongly affects the user’s impression (e.g. likeability) of the agent; a lower performance than expected leads to an unlikable impression^21,22^. This suggests that the changes in mind perception during a task might influence the attribution process. Thus, lower Agency and Experience than expected before a task might lead to an unfavorable impression of the agent, which induces the following hypotheses:

### Hypothesis 3

A decrease in Agency during a task increases causal attribution to an agent.

### Hypothesis 4

An increase in Experience during a task increases responsibility attribution to an agent.

H3 and H4 consider changes in mind perception before and after the game, while H1 and H2 consider mind perception at the end of the game. To validate H1 and H2, we analyze the relationships between the post-game mind perception scores and causal and responsibility attribution. To validate H3 and H4, we use “pre-post mind perception” by subtracting the pre-game mind perception scores from the post-game scores.

## Method

### Participants

Fifty Japanese participants were recruited through a participant recruitment agency for a fee of 5000 yen per participant. If the partner was human, we introduced to the participant that the partner was another participant. However, two participants noticed that the human agent was an experimenter. Therefore, those two participants were excluded from the data analysis. Thus, we analyzed data for 48 participants (24 female), aged 20 to 29 years (M = 23.5, SD = 2.4). Before the experiment, each participant was instructed that the amount of a monetary reward depended on the game’s result. All participants completed the games and questionnaires in all three agent conditions, using a within-subject design. The experimental order of the three agents was randomized for each participant. After the experiment, the purpose of the experiment was explained to the participants again, a fixed amount of 1000 yen on top of the 5000 yen was given to them regardless of the outcome of the game, and their consent was obtained again.

### Game design^19^

**Figure 1:**
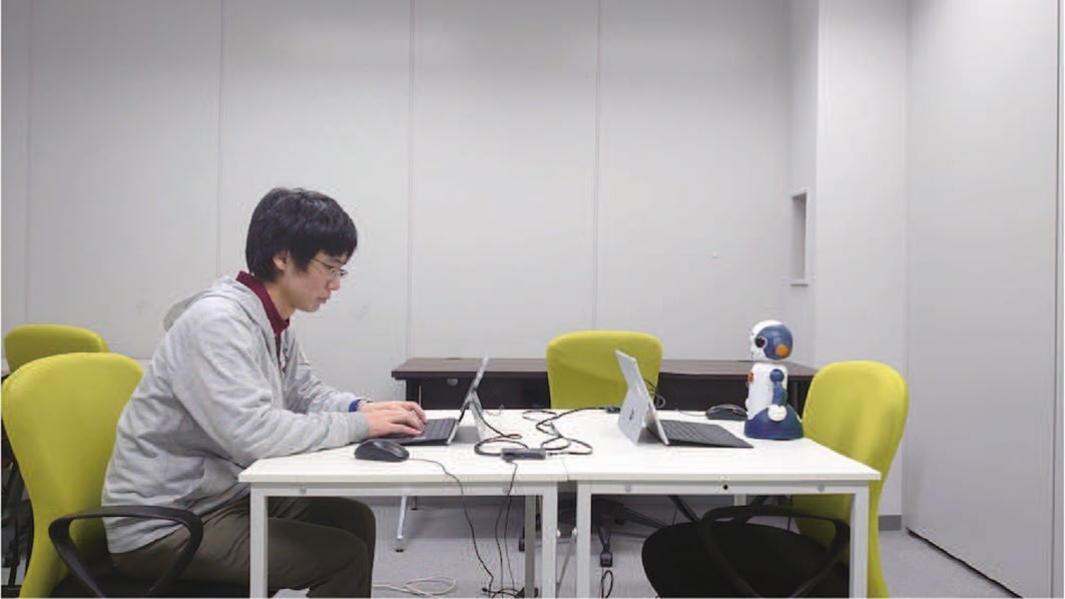
Scene of the experiment in the robot condition.

Participants played the game with each agent using a computer (Fig. 1 for the robot condition). This game was a repeated noncooperative game, similar to the prisoner’s dilemma, where a participant and an agent respectively choose one of two options: “I want more” or “I will give it over to my partner.” The amount of the monetary reward was decided in accordance with their combined choices. The rules of the reward or payoff are described below (see Table 1 for summary).

**Table 1:** Payoff table.

		Participant	
		I want more.	I will give it over to my partner.
Agent	I want more.	Participant: $-10$ yen Agent: $-10$ yen	Participant: $+20$ yen Agent: $+100$ yen
	I will give it over to my partner.	Participant: $+100$ yen Agent: $+20$ yen	Participant: 0 yen Agent: 0 yen

- If a participant and agent choose“I want more” and “I will give it over to my partner,” respectively, then they obtain 100 yen and 20 yen, respectively (bottom left in Table 1).
- If a participant and agent choose “I will give it over to my partner” and “I want more,” respectively, then they obtain 20 yen and 100 yen, respectively (top right in Table 1).
- If both a participant and agent choose “I will give it over to my partner,” then they cannot obtain a monetary reward (bottom right in Table 1).
- If both a participant and agent choose “I want more,” then they lose 10 yen (top left in Table 1).

This payoff matrix was always displayed on the computer screen during the game. The participant could see his or her own decision; however, he or she could not see the agent’s decision during the decision-making process. The outcome and agent’s decision appeared after decisions were made. This game was repeated ten times in each trial. The total sum of rewards for the ten games was given to the participant. Participants were informed of a false mean reward (500 yen) as the amount of money averaged over past participants before the game, in order to set participants up with a prior expectation. Participants were instructed not to talk to the partner agent during the game.

### Agents^19^

**Figure 2:**
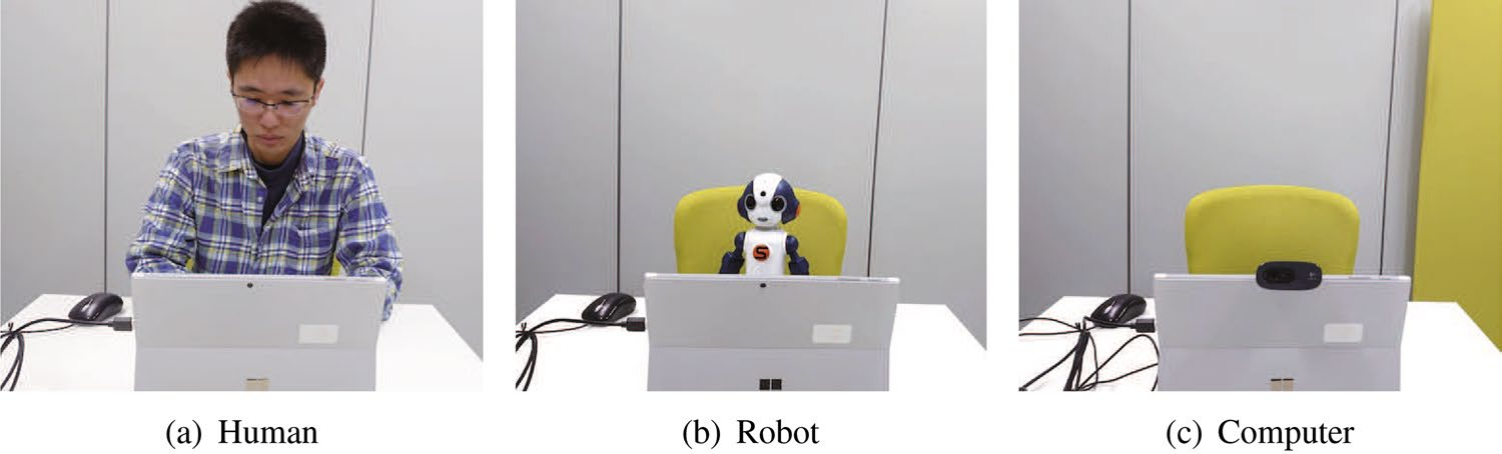
Partner agents.

We designed three conditions for the partner agent: a human (a woman aged 25 years), robot (Sota, Vstone Co., Ltd.), and computer (Surface, Microsoft; Fig. 2a–c, respectively). All participants played the ten-game trial once with each agent. The partner agents chose “I want more” six times and “I will give it over to my partner” four times in a random order, regardless of the participant’s choice. The order of the agents’ choices was also randomized for each trial. Due to the random choices, participants’ total rewards were always less than the false total rewards (500 yen).

Before the game, the capabilities of the agents were explained as follows: 

Human condition: This agent was introduced to participants as another participant who was playing the same game in another room. In this condition, the agent as well as the participant answered the questionnaires described in the next section, both before and after the game, to make the participant believe the agent was a naive participant.

Robot condition: Participants were instructed that this robot used artificial intelligence developed at Osaka University and could make decisions based on a participant’s facial expressions observed by a camera in the robot’s eyes and that the money obtained by the robot would be used to develop it. After this instruction, the robot nodded and stated, “Nice to meet you; I will do my best.” During the game, the robot slightly moved its neck at random when idle.

Computer condition: We mounted a web camera on the computer so that participants can see the eye (a camera) of the partner agent, as with the human and robot agents. Participants were instructed that this computer used artificial intelligence developed at Osaka University and could make decisions based on a participant’s facial expressions observed by the web camera and that the money obtained by the computer would be used to develop it. The agent neither moved nor spoke in this condition.

### Measurement

Each participant evaluated the mind perception^15^ (Japanese version of the questionnaire^23^) of the partner agent before the game. After the game, the participant remained seated and again evaluated the mind perception of the partner agent. The 18 items of the mind perception questionnaire are listed in Table 3. The questionnaires were scored using a 7-point Likert scale (1 = strongly disagree; 7 = strongly agree). For example, the question about “memory” was “how capable is the partner of memorizing something?”

After the game, participants answered the following items about causal attribution (a and b) and responsibility attribution (c and d) for the game’s outcome for themselves or the partner agents.

- The partner was the cause of your reward falling below the average (500 yen).
- You were the cause of your reward falling below the average (500 yen).
- The partner was responsible for your reward falling below the average (500 yen).
- You were responsible for your reward falling below the average (500 yen).

The questions were scored using a 7-point Likert scale (1 = strongly disagree; 7 = strongly agree). We defined relative causal attribution as the value obtained by subtracting the score of (b) from that of (a). Similarly, relative responsibility attribution was defined by subtracting the score of (d) from that of (c). These respectively represented how much a participant attributed the cause and responsibility to the partner agent compared to himself or herself. All questionnaires were rated on a seven-point scale.

### Analyses

First, a factor analysis was used to extract the “Experience” and “Agency” factors from the rating scores of the 18 items of the mind perception questionnaire. Factor analysis is a statistical method which explains the variability among observed variables in terms of fewer unobserved variables called factors. We performed the factor analysis with the maximum likelihood method and promax rotation for the scores of post-game mind perception questionnaires for all agent conditions. We regarded factors with eigenvalues over 1.0 as principal factors. Then, the post-game and pre-game rating scores were mapped into the principal factors using the estimated factor loadings.

Next, using SEM analysis, we investigated the relationships between the factor scores and relative causal and responsibility attribution scores. SEM analysis is a statistical modeling technique used to analyze the relationships among variables. It provides a way to test and estimate causal relationships between variables and to examine the overall fit of a model to observed data. The first step is to assume a theoretical model as a system of linear equations that represent the relationships among variables. A path diagram is usually used to depict the model, where rectangles, circles, and paths (arrows) indicate observable variables, latent variables, and linear relationships between variables, respectively. We constructed a model for each agent based on our hypotheses and results of correlation analysis. The next step is to estimate the model parameters, i.e. values of regression coefficients and error variances that best fit the observed data. Then, we assessed the models’ fit to the data using fit indices such as goodness-of-fit index (GFI), adjusted GFI (AGFI), normed fit index (NFI), comparative fit index (CFI), root mean square error of approximation (RMSEA), and Akaike’s information criteria (AIC). Finally, we modified the models (removed paths) to improve the fit.

### Procedure

We explained the purpose and the procedure of the experiment to the participants before the experiment. This study was approved by the ethics committee for research involving human subjects at the Graduate School of Engineering, Osaka University. All methods were performed in accordance with relevant guidelines and regulations. We provided instructions for the experiment after obtaining signed informed consent from all participants. First, participants practiced the game once with an experimenter to confirm they understood how to play. Then, participants faced an agent and were instructed on the agent’s capability. Participants evaluated the agent using the mind perception scale, then played the game. After the game, participants evaluated the agent using the same mind perception scale and attributions of cause and responsibility for the failure. The games and evaluations for the other agents were then conducted using the same method. During agent switching, participants waited outside the experiment room.

## Results

### Causal and responsibility attributions

**Figure 3:**
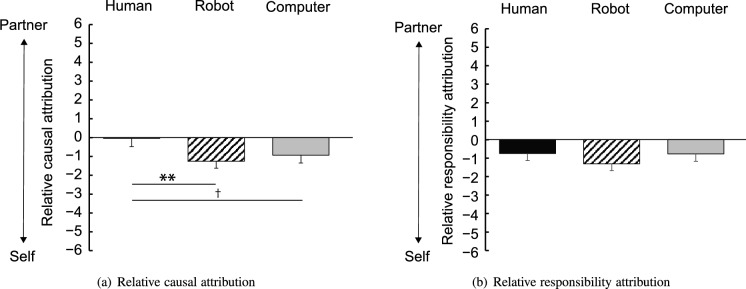
Relative attributions of cause (**a**) and responsibility (**b**). This value means the degree of attribution to the partner agent compared with one’s self. Error bars indicate standard error. ($$\dag $$$$p < 0.10$$; $$^*$$$$p < 0.05$$; $$^{**}$$$$p < 0.01$$).

Table 2 lists the scores of causal and responsibility attributions averaged across participants. One-way ANOVAs indicated no significant main effects of the agents for causal attribution to the partner agents ($F(2, 140) = 2.27$, $p = 0.10$, $\eta _p^2 = 0.031$), for causal attribution to himself or herself ($F(2, 140) = 1.20$, $p = 0.30$, $\eta _p^2 = 0.017$), for responsibility attribution to the partner agents ($F(2, 140) = 0.64$, $p = .64$, $\eta _p^2 = 0.009$), or for responsibility attribution to himself or herself ($F(2, 140) = 0.68$, $p = 0.51$, $\eta _p^2 = .010$). Figure 3 shows the scores of (a) relative causal attribution and (b) relative responsibility attribution obtained by subtracting the scores of attributions to himself or herself from the scores of attributions to the agents. One-way ANOVAs did not indicate any significant main effects of agents in either relative causal attribution ($F(2, 140) = 2.42$, $p = 0.09$, $\eta _p^2 = .033$) or relative responsibility attribution ($F(2, 140) = 0.70$, $p = 0.51$, $\eta _p^2 = 0.010$). *Post-hoc* paired *t*-tests with Bonferroni corrections elucidated a significant difference in relative causal attribution between the human condition and robot condition ($t(47) = 2.94$, $p = .005$).

**Table 2:** Mean (SD) scores of causal and responsibility attributions.

		Human	Robot	Computer
Causal	Partner	4.08 (1.72)	3.42 (1.62)	3.42 (1.93)
	Self	4.13 (1.75)	4.67 (1.59)	4.35 (1.85)
Responsibility	Partner	3.42 (1.75)	3.02 (1.73)	3.10 (1.93)
	Self	4.17 (1.93)	4.33 (1.93)	3.88 (2.09)

When the robot and computer conditions were combined into an artificial condition, a significant difference in relative causal attribution was observed between the artificial condition ($M=- 1.09$, $SD=2.26$) and the human condition ($t(142) = 2.13$, $p = 0.03$). This result indicated that less causal attribution was made to the artificial agents than the human agent. For relative responsibility attribution, the difference between the artificial condition ($M=- 1.04$, $SD=2.21$) and the human condition was not significant ($t(142) = 0.63$, $p = 0.52$).

### Factor analysis for mind perception

We performed a factor analysis for the rating scores of the post-game mind perception questionnaires. We found two factors with eigenvalues over 1.0: the first factor corresponding to “Experience” (eigenvalues $= 9.59$, accounted for 43% of the variance) and the second factor corresponding to “Agency” (eigenvalues $= 2.12$, accounted for 19% of the variance). Table 3 presents detailed factor loadings. This factor structure was similar to that proposed by Gray et al.^15^, except that “morality” belonged to Experience rather than Agency in the current experiment.

**Table 3:** Factor loadings.

Mental capacity	1st factor experience	2nd factor agency
Pain	$\mathbf {1.026}$	$- 0.203$
Fear	$\mathbf {1.006}$	$- 0.191$
Hunger	$\mathbf {0.928}$	$- 0.189$
Rage	$\mathbf {0.878}$	$- 0.040$
Pleasure	$\mathbf {0.875}$	0.001
Pride	$\mathbf {0.837}$	0.003
Joy	$\mathbf {0.761}$	0.130
Desire	$\mathbf {0.712}$	0.121
Personality	$\mathbf {0.609}$	0.169
Embarrassment	$\mathbf {0.582}$	0.218
Morality	$\mathbf {0.560}$	0.217
Planning	$- 0.257$	$\mathbf {0.914}$
Thought	$- 0.107$	$\mathbf {0.855}$
Memory	$- 0.276$	$\mathbf {0.751}$
Self-control	$- 0.006$	$\mathbf {0.613}$
Emotion recognition	0.214	$\mathbf {0.453}$
Communication	0.360	$\mathbf {0.451}$
Consciousness	0.391	$\mathbf {0.444}$

**Figure 4:**
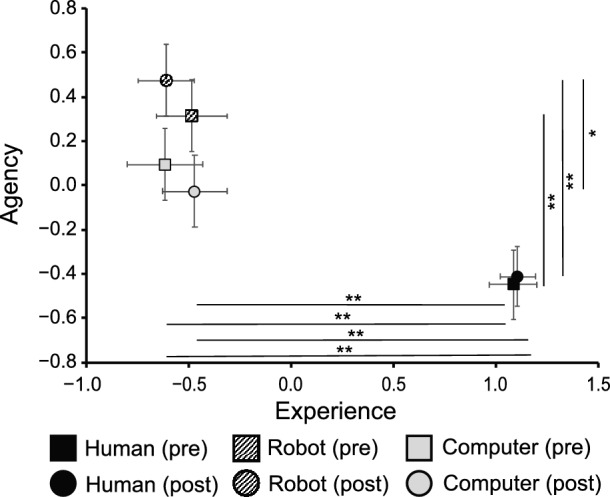
Mind perception in two dimensions. The square and round markers indicate mind perception before and after the game, respectively. Error bars indicate standard error. ($$^*$$$$p < 0.05$$; $$^{**}$$$$p < 0.01$$).

Figure 4 shows the post-game mind perception space to which the scores before and after the game were mapped. A two-way ANOVA (agent $\times$ pre-post) for Experience indicated that a main effect of agent was significant ($F(2, 276) = 14.7$, $p < 0.001$, $\eta _p^2 = 0.096$) while a main effect of pre-post ($F(1, 276) = 0.14$, $p = 0.71$, $\eta _p^2 = 0.001$) and an interaction effect ($F(2, 276) = 0.04$, $p = 0.99$, $\eta _p^2 = 0.000$) were not significant. *Post-hoc* paired *t*-tests with Bonferroni corrections elucidated that the Experience score of the human agent before the game was significantly greater than those of the robot and computer agents ($t(47) = 8.55, p < 0.001$ and $t(47) = 8.22, p < 0.001$, respectively), and the Experience score after the game was also similar ($t(47) = 9.04, p < 0.001$ and $t(47) = 6.86, p < 0.001$, respectively). A two-way ANOVA (agent $\times$ pre-post) for Agency also indicated a significant main effect of agent ($F(2, 276) = 4.31$, $p = 0.014$, $\eta _p^2 = 0.030$) while it did not indicate a significant main effect of pre-post ($F(1, 276) = 14.7$, $p = 0.54$, $\eta _p^2 = 0.001$) or a significant interaction effect ($F(2, 276) = 0.01$, $p = 0.99$, $\eta _p^2 = 0.000$). *Post-hoc* paired *t*-tests with Bonferroni corrections elucidated that the Agency score of the robot agent before the game was significantly greater than that of the human agent ($t(47) = 3.69, p = 0.002$), and the Agency score of the robot agent after the game was significantly greater than those of the human and computer agents ($t(47) = 4.09, p < 0.001$ and $t(47) = 2.06, p = 0.037$, respectively). These results indicate that the human partner was perceived as an agent with more Experience and less Agency than the artificial agents.

The post-game factor scores were used as “post-Experience” and “post-Agency” scores in the subsequent analyses. Additionally, we used the pre-post differences in Experience and Agency as “pre-post Experience” and “pre-post Agency” scores, that were obtained by subtracting the pre-game factor scores from post-game factor scores.

### Correlation analysis

Table 4 lists the correlation coefficients between relative causal and responsibility attribution scores and mind perception scores: “post-Experience,” “post-Agency,” “pre-post Experience,” and “pre-post Agency.” We conducted two-tailed one-sample *t*-tests for these correlations in which a false discovery rate method was applied. Very strong correlations were found between relative causal and responsibility attributions in all conditions (all *p*s $< 0.001$). We found different significant correlations for the mind perception between the agents. In the human condition, relative causal attribution significantly correlated with pre-post Agency (r = – 0.358, $p = 0.013$). This correlation was negative, indicating that the large decreases in Agency through the game were more related to causal attribution to the human agent. In the robot condition, relative causal attribution significantly negatively correlated with post-Agency ($r = - 0.400$, $p = 0.005$), indicating that the robot agent who the participant considered to have agency tended not to be the attributed cause. The relative responsibility attribution had a significant positive correlation with post-Experience ($r = 0.340$, $p = 0.018$) and a significant negative correlation with post-Agency ($r = - 0.429$, $p = 0.002$) in the robot condition. This indicated that more Agency and less Experience after the game related to less responsibility attribution to the robot. There were no significant correlations between relative attribution and mind perception in the computer condition. We did not find any significant correlations between pre-game mind perception scores and relative attribution scores in all conditions.

**Table 4:** Correlation coefficients.

		Relative causal attribution	Relative responsibility attribution
Human			
Post	Experience	– 0.092	– 0.095
	Agency	– 0.315	– 0.257
Pre-post	Experience	0.172	0.162
	Agency	– 0.$358^{*}$	– 0.301
Relative causal attribution		–	0.$$754^{***}$$
Robot			
Post	Experience	0.177	$0.340^*$
	Agency	$$- 0.400^{**}$$	$$- 0.429^{**}$$
Pre-post	Experience	0.027	0.045
	Agency	– 0.143	– 0.162
Relative causal attribution		–	$$0.810^{***}$$
Computer			
Post	Experience	0.137	0.284
	Agency	– 0.116	– 0.233
Pre-post	Experience	0.099	0.288
	Agency	– 0.176	– 0.274
Relative causal attribution		–	$$0.750^{***}$$

($$^*$$$$p < 0.05$$, $$^{**}$$$$p < 0.01$$, $$^{***}$$,$$p < 0.001$$. A false discovery rate method was applied)

The scores of relative causal and responsibility attributions did not significantly correlate with the total amount of participants’ rewards, total amount of partners’ rewards, or the number of times that the partner chose “I want more” in each agent condition, even without applying any multiple comparison correction methods (all *p*s $> 0.10$). See Table 6 in Appendix A for their correlation coefficients.

### SEM analysis

For SEM analysis, we constructed a tentative full model from mind perception to responsibility attribution based on our hypotheses and correlation analysis results, then explored the best fitted model for each agent to verify our hypotheses. We assumed that causal attribution to an agent leads to responsibility attribution to the agent, which is based on a basic attribution theory that people attribute more responsibility for an event to the actor who causes the event^4^. Then, paths from post-Agency and pre-post Agency to relative causal attribution were assumed according to Hypotheses 1 and 3, that Agency affects causal attribution. Additionally, we assumed paths from post-Experience and pre-post Experience to relative responsibility attribution based on Hypotheses 2 and 4, that Experience affects responsibility attribution. The model did not include paths of pre-Experience, pre-Agency, or other game outcomes (e.g. the reward amount) because they did not have any significant correlations with relative attribution scores.

**Figure 5:**
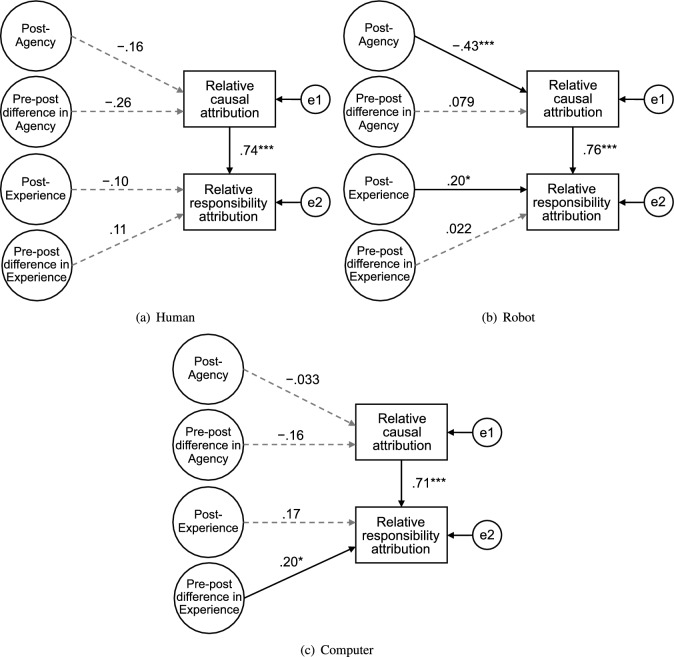
The full structural equation model from mind perception to responsibility attribution for the (**a**) human, (**b**) robot, and (**c**) computer conditions. The paths with significant and nonsignificant coefficients are represented by solid and broken arrows, respectively. ($$^*$$$$p < 0.05$$, $$^{**}$$$$p < 0.01$$, $$^{***}$$$$p < 0.001$$).

Figure 5 shows the SEM analysis results using the full model and depicts the standardized coefficients for all paths. Statistically significant and nonsignificant paths are indicated as solid and broken arrows, respectively. Fit indices of the model for each agent are listed in Table 5. There were strong paths from relative causal attribution to relative responsibility attribution in all conditions ($p < 0.001$). In the human condition, we did not find significant paths from mind perception to relative attributions; however, the model showed relatively poor fit. Contrastingly, the paths from post-Agency to relative causal attribution and from post-Experience to relative responsibility attribution were significant in the robot condition ($p = 0.005$ and $p = 0.015$, respectively). Only the path from pre-post Experience to relative responsibility attribution was significant in the computer condition ($p = 0.023$).

**Figure 6:**
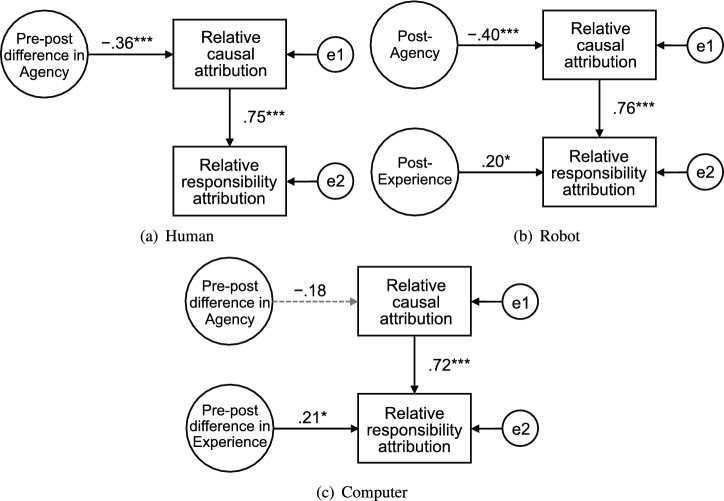
The simplified best fit structural equation models from mind perception to responsibility attribution for the (**a**) human, (**b**) robot, and (**c**) computer conditions. The paths with significant and nonsignificant coefficients are represented by solid and broken arrows, respectively. ($$^*$$$$p < 0.05$$, $$^{**}$$$$p < 0.01$$, $$^{***}$$$$p < 0.001$$).

**Table 5:** Fit indices for the full and simplified structural equation models for each agent.

Model	Agent	$\chi ^2 /$ df	GFI	AGFI	NFI	CFI	RMSEA	AIC
Full	Human	2.628	0.937	0.671	0.932	0.954	0.415	24.511
	Robot	1.026	0.972	0.855	0.980	0.999	0.037	18.103
	Computer	0.458	0.987	0.934	0.987	1.000	0.000	15.832
Simplified	Human	0.122	0.998	0.990	0.997	1.000	0.000	8.123
	Robot	0.336	0.993	0.965	0.993	1.000	0.000	10.671
	Computer	0.204	0.996	0.979	0.996	1.000	0.000	10.408

*GFI* goodness-of-fit index, *AGFI* adjusted GFI, *NFI* normed fit index, *CFI* comparative fit index, *RMSEA* root mean square error of approximation, *AIC* Akaike’s information criteria.

Figure 6 shows the simplified model exhibiting the best fit for each agent (see Table 5 for fit indices). The model for the human agent had a significant path from pre-post Agency to relative causal attribution ($p = 0.009$). This path was consistent with the correlation analysis results. The model for the robot agent consisted of the paths from post-Agency to relative causal attribution ($p = 0.003$) and from post-Experience to relative responsibility attribution ($p = 0.022$), which were the same as the significant paths in the full model and consistent with the significant correlations. In the computer condition, the path from pre-post Experience to relative responsibility attribution was significant ($p = 0.019$); however, this relation was not supported in the correlation analysis.

## Discussion

The experimental results showed the relation between specific mind perception and causal and responsibility attributions. We found consistent significant effects between the results of the correlation analysis (see Table 4) and SEM analysis (see Fig. 6); a decrease in Agency during the game increased causal attribution to the human agent, Agency after the game decreased causal attribution to the robot agent, and Experience after the game increased responsibility attribution to the robot agent. Notably, objective game outcomes, such as amounts of participants’ or agents’ rewards or the number of times the agent selected “I want more” were not related to causal and responsibility attributions (see Table 6 in Appendix A). The results suggested that attribution to the partner agent was affected by subjective mind perception regarding the agent and a gap from prior expectations rather than objective monetary damage at least in the current experiment, dealing with a small amount of money. Therefore, robot designers should consider not only a robot’s task performance but also a user’s mind perception regarding the robot. Further, we found different attribution processes from mind perception to responsibility attribution among the agent types. We will discuss Hypothesis 1–4 for each agent in the following sections.

### Hypothesis 1: post-agency decreases causal attribution

Hypothesis 1 was accepted in the robot condition but rejected in the human and computer conditions. Existing studies have shown that agency perception increases responsibility attribution to a robot for outcomes of the robot’s actions^8,15,18^. However, in the current experiment where an outcome’s cause was vague as to whether it was attributable to one’s self or the robot, thus leading to lower responsibility attribution to the robot. The Agency dimension corresponds to machine intelligence^16^ and consists of the important capabilities for the game’s success (e.g. memory and planning). Therefore, participants might have evaluated the robot as having high Agency affecting the success of the game, which in turn might have led participants to attribute the cause to themselves more than to the capable robot. However, the computer’s Agency did not affect causal attribution possibly, while the number of times the participant chose “I want more” and the partner’s total reward relatively correlated with causal attribution even though they were not statistically significant ($r=- 0.18$, $p= 0.21$ and $r= 0.23$, $p= 0.12$, respectively, see Table 6). This implies a possible interpretation that participants might consider some objective game outcomes when they attribute a cause to a computer.

### Hypothesis 2: post-experience increases responsibility attribution

Hypothesis 2 was accepted in the robot condition but rejected in the human and computer conditions. Participants attributed more responsibility to the robot they perceived as having emotional capabilities. People might be more sensitive to robots having emotions than to humans and computers. The classical attribution theory holds that people attribute responsibility for the outcome of an agent’s action as being motivated by its emotion or internal intention^4,5^. Therefore, people might think that the robot should be held responsibility for its selfish behavior according to its own emotions. In addition, the uncanny valley of mind has been reported in which perceiving a nonhuman agent to have emotional capabilities makes one feel uncanny about it^24,25^. Such negative feelings toward the robot might bias the increased responsibility attribution^20^. This may suggest that the robot’s emotion should be carefully designed when considering responsibility for a failure as attributed to the robot. Such an effect was not observed in the computer condition, possibly because humans do not expect emotions to affect a computer’s decisions.

### Hypotheses 3 and 4: pre-post differences in mind perception affect attribution

Hypothesis 3 was accepted only in the human condition, while Hypothesis 4 was rejected in all conditions. The participants inferred more causality for a human partner whom they perceived as having less Agency than prior expectations. This might be because participants expected a human to make an altruistic decision for the game’s success (i.e. “I will give it over to my partner”). However, the expected choices were not actually made, which might have led to more causal attribution. Contrastingly, this gap effect was not observed in the robot and computer conditions, possibly because such behavior was not expected for them. Malle et al.^26^ showed that people expect moral decisions and utilitarian decisions for humans and robots, respectively, in a moral dilemma. Therefore, the expectation of actions to be taken by human and artificial agents are different, and betrayal of those expectations might lead to large causal attribution to the human agent. The adaptation gap hypothesis, that inferior actual performance of an agent to prior expectation leads to the unfavorable impression of the agent^21,22^, might underlie this process.

The effect of the pre-post gap in Agency rather than post-Agency was observed in the human condition, because participants might have prior expectations for the human agent. Contrastingly, since they might not expect a robot to make a successful decision, only post-Agency related to causal attribution to the robot. There were no consistent significant relations between mind perception and attributions in the computer condition. This might have been because participants did not regard the computer as an anthropomorphic agent in this experiment, thus implying that apparent human-likeness might be a trigger for the anthropomorphic-based attribution process.

### Limitations and future directions

Possible cultural differences in attribution should be considered, as all participants in our experiment were Japanese. It has been reported that Japanese people tend to attribute the cause of a failure to themselves, in a so-called self-effacing bias^27,28^, and the fact that they tend to attribute more Agency to artificial agents was found by Gray et al.^15,16^. Therefore, people in countries other than Japan could tend to attribute the cause and responsibility to a robot more than themselves. Additionally, individual differences, such as negative attitudes toward robots^29^ and previous experiences using robots, might affect attribution to robots.

In this study, we conducted experiments under three conditions in which the degree of anthropomorphism would vary: a human, robot, and computer. These agents are common entities that interact with people in the real world. However, it is necessary to investigate attributions using a variety of agents, including more human-like android robots, to generalize the attribution models in this study.

The monetary damage was minor for participants (i.e. the loss of several hundred yen or a few dollars) in this experiment. If a failed outcome is more serious (e.g. the loss of a large amount of money (a few hundred dollars or a few percent of the person’s income) or a person’s death), causal and responsibility attributions to the agent might increase. A classic psychological study showed that people attributed more responsibility for actions to an actor when the outcome was more serious^30^. Thus, future research should study how mind perception affects attribution in such serious situations.

With regard to the above point, this experiment used a simple game, however, it is necessary to conduct experiments in a more realistic situation. For example, negotiations with a shopkeeper robot or decision making on the road with a mobile robot could be considered. If such experiments can be conducted, the results could be more generalized, leading to a design theory for robots that avoids overly biased attributions of cause and responsibility.

We evaluated participants’ responsibility attribution using a simple question: “Are you or the partner responsible for your reward falling below the average?” However, the concept of responsibility includes pure causation, apology, blame, punishment, compensation, accountability, etc., and what kind of responsibility is appropriate and necessary might differ by agent types. Therefore, in future research, we plan to investigate responsibility attribution in detail, to further distinguish aspects of the concept of responsibility. What type of responsibility should be attributed to robots might depend on the robot’s abilities, such as to feel pain^31^ or to possess property.

A noncooperative game was used in this study because we supposed human-robot economical interactions (e.g. a robot in a shop), where cooperation between a human and agent was not explicitly assumed. Situations in which humans and robots have the same goal and cooperate with each other are assumed in many social interactions with robots. Such situations can be applied not only to joint decision-making with a machine but also to shared control systems^32^. For example, a semi-automatic driving system needs to cooperate with a human driver to control a car^33^, and a wearable power assist robot and its user share control of the user’s body^34^. In shared control, the human infers or feels whether he/she is controlling the action or the machine is, which is referred to as the sense of agency^35^. The mechanism of the sense of agency and its relationship with subjective responsibility is also starting to be studied in neuroscience^36^. Using interdisciplinary approaches including not only social psychology but also engineering and neuroscience, we would like to study the cognitive process of causal and responsibility attribution in the future.

## Conclusion

We investigated the relationship between mind perception composed of Experience (emotion) and Agency (intelligence) dimensions and attributions of the cause and responsibility for failing a game against an agent. We obtained SEM results from mind perception to responsibility attribution, which differ by agent type: human, robot, or computer. In the human model, a decrease in Agency during the game led to causal attribution to the human partner, which resulted in responsibility attribution to the human partner. In the robot model, Agency perception after the game decreased the degree of causal attribution to the robot, and the degree of responsibility attribution to the robot was based on the causal attribution and was increased by Experience perception after the game. There were no significant relationships between mind perception and causal and responsibility attributions in the computer model. This result introduced the perspective of the anthropomorphic process into the existing attribution theory and could lead to a design theory for the appropriate degree of attribution in human-machine interactions. Further, our approach may offer experimental evidence to the philosophical discussion regarding artificial moral agency^37,38^.

## Data Availability

The datasets used and/or analysed during the current study available from the corresponding author on reasonable request.

## Appendix A: A Correlation coefficients with the game outcome

Table 6 lists correlation coefficients between relative causal and responsibility attributions and the game outcomes in each agent condition. They were not statistically significant.

**Table 6:** Correlation coefficients between the relative attribution scores and game outcomes.

	Relative causal attribution	Relative responsibility attribution
Human		
The number of times a participant chose “I want more”	0.048	0.075
Participant’s total reward	0.129	0.127
Partner’s total reward	0.105	0.051
Robot		
The number of times a participant chose “I want more”	0.077	– 0.113
Participant’s total reward	– 0.073	– 0.100
Partner’s total reward	– 0.120	0.037
Computer		
The number of times a participant chose “I want more”	– 0.183	– 0.166
Participant’s total reward	– 0.053	– 0.014
Partner’s total reward	0.230	0.183
